# Food Consumption during Binge Eating Episodes in Binge Eating Spectrum Conditions from a Representative Sample of a Brazilian Metropolitan City

**DOI:** 10.3390/nu15071573

**Published:** 2023-03-24

**Authors:** Carlos Eduardo Ferreira de Moraes, Marina Maria Leite Antunes, Carla Mourilhe, Rosely Sichieri, Phillipa Hay, Jose Carlos Appolinario

**Affiliations:** 1 Group of Obesity and Eating Disorders (GOTA), Psychiatry Institute (IPUB), Federal University of Rio de Janeiro, Rio de Janeiro 21941-901, Brazil; 2 Translational Health Research Institute, School of Medicine, Western Sydney University, Sydney, NSW 2750, Australia; 3 Department of Epidemiology, Social Medicine Institute (IMS), State University of Rio de Janeiro (UERJ), Rio de Janeiro 28625-570, Brazil; 4 Mental Health Services, South West Sydney Local Health District (SWSLHD), Campbelltown, NSW 2560, Australia

**Keywords:** Binge Eating Disorder, Bulimia Nervosa, recurrent binge eating, energy intake, macronutrients, food consumption

## Abstract

The prevalence of binge eating spectrum conditions (BESC) are increasing globally. However, there is a lack of data from general population samples in low- and middle-income countries. Thus, this study described the food consumption during objective binge eating episodes (OBE) in people with BESC from a metropolitan city in Brazil. Participants comprised 136 adults (18 years old–60 years old) with Binge Eating Disorder (BED), Bulimia Nervosa (BN), or recurrent binge eating (RBE) from a two-phase epidemiological survey. They were interviewed in their homes by trained lay interviewers using the Questionnaire on Eating and Weight Patterns updated for the DSM-5 to assess BESC diagnosis and food consumption during a typical OBE. Overall, participants consumed a mean of 1067 kcal during the episodes. For the most part, these calories were derived from carbohydrates (58%) and lipids (30%), irrespective of the diagnosis. Regarding food item consumption, individuals with BED and RBE consumed staple foods (mainly rice and beans) more frequently than those with BN. Conversely, participants with BN ingested sugar-sweetened beverages more frequently than the BED group. In conclusion, there were differences in the eating patterns of individuals with BESC in Brazil. BED and RBE participants consumed more typical foods, whereas those with BN preferred foods with a high content of energy during their OBE.

## 1. Introduction

Objective binge eating episodes (OBE) are characterized by the consumption of excessive amounts of food in a discrete time interval (<2 h) in which individuals experience a feeling of loss of control over eating [1]. It is a core feature in the psychopathology of Binge Eating Disorder (BED) and Bulimia Nervosa (BN) [2]. According to the Diagnostic and Statistical Manual for Mental Disorders, fifth edition (DSM-5), both conditions are characterized by regular OBE that occurs weekly in the most recent three months [2]. However, to fulfill the diagnosis of BED, individuals should report at least three of the five of the following behaviors associated with loss of control: eating fast, eating until feeling disconformably full, eating large amounts of food when not feeling hungry, eating alone due to feeling embarrassed by the amount of food eaten, and feeling guilty or depressed after the episode [2]. Furthermore, although people with BED feel marked distress regarding OBE, they do not engage in inappropriate compensatory behaviors (e.g., self-induced vomiting, fasting, misuse of laxatives/diuretics). BED is commonly associated with higher Body Mass Index (BMI) but is also diagnosed in people within the healthy range [3]. Conversely, in BN, the OBE are followed by recurrent inappropriate compensatory behaviors to avoid weight gain [2]. Additionally, only this condition requires the overvaluation of weight and shape or body image disturbances to fulfill the diagnosis [2]. It is noteworthy that the presence of marked distress regarding OBE and behaviors associated with loss of control are not included in the BN diagnostic criteria. In addition, the presence of BN is uncommon among individuals with BMI ≥ 30 kg/m^2^. Taken together, BED and BN are the most common eating disorders (ED) worldwide [4]. There has been a doubling of their global prevalence within the past two decades [5], reaching more than 5% in community samples [6]. Furthermore, these EDs are associated with clinical comorbidities and significant impairment in functioning and quality of life [6,7,8,9].

The construct of binge eating spectrum conditions (BESC) is increasingly employed in the literature and encompasses threshold forms of ED (BED and BN) and also partial syndromes such as recurrent binge eating (RBE) [10,11,12]. RBE is conceptualized as episodes of binge eating occurring at least on a weekly basis, but not fulfilling criteria for a full ED diagnosis. Although not recognized as a formal category of ED, such people with RBE have been determined to have clinical and functional impairment [13,14,15], and such cases are reported to be increasing in prevalence. For example, in Germany and in Western Asia, the point prevalence was 0.9 and 3.5, respectively [16,17]. In the Australian population, the prevalence of RBE increased from 2.8% in 1998 to 13% in 2015 [15]. Further, this increase was greatest in those with high weight—another major public health burden [14]. Additionally, the distress related to RBE was associated with higher impairment in health-related quality of life [15]. In Brazil, Freitas et al. determined a prevalence of 11.5% in the previous 6 months in a general population sample of middle-aged women from the city of Rio de Janeiro [13]. Similarly, in a Brazilian National cohort, the prevalence of RBE was 15.3% in the same timeframe [18]. Furthermore, people with RBE were at a higher risk of hypertension and hypertriglyceridemia [18].

Information on the prevalence of BESC originates mostly from high-income countries [5]. However, the population-based data from middle-income countries indicates that those conditions are similarly prevalent and impairing [6,9,19]. For instance, in Brazil, data from representative samples of two metropolitan cities revealed that the point prevalence of BED and BN was around 1.5% and 0.8%, respectively [6,9]. However, the lifetime prevalence was 4.7% for BED and 2% for BN [6,9]. Regarding RBE, data from Brazilian epidemiological studies indicate that this disordered eating behavior is commonly reported in the community, with point prevalence ranging from 6.2% to 11.5% [9,13]. Further, it has been associated with higher Body Mass Index (BMI), clinical and psychiatric comorbidities, and functional impairment [9,13]. Taken together, these findings also highlight the public health impact of BESC in middle-income countries.

Although it has been described since the 1950s, there is still debate regarding some features of OBE [1]. For example, there is a lack of a standardized definition for “an excessive amount of food” [20,21]. A recent systematic review with meta-analysis of 43 studies reported that individuals with BED consumed 2088 kcal during OBE in clinical studies and 1903 kcal in laboratory settings [20]. Regarding BN, the authors determined that the amount of calories consumed during OBE was 1789 kcal in clinical studies but much more than people with BED, i.e., 3070 kcal, in laboratory settings [20]. However, more than 90% of the studies included were performed in high-income countries (e.g., United States) [20]. In addition, there was only one study comparing the dietary intake during the episodes across ED diagnoses. Fitzgibbon et al. determined that people with BN consumed more calories from carbohydrates than those with BED [22]. Regarding the food preferences during OBE, laboratory studies show that individuals with BED frequently eat dairy foods, sweets, snacks, and meats [23,24,25]. Similarly, clinical studies revealed that people with BN consume sweets/desserts, snacks, and dairy food more frequently during the episodes [26,27,28].

Data on the food consumption during OBE originated mostly from studies performed in laboratory or clinical settings. These are inherently subject to selection biases; for example, people in clinical settings with ED may have more complex or severe symptoms and what is eaten in the laboratory may not reflect what is consumed in the person’s everyday life. In addition, to our knowledge, there is no information on the food consumption during OBE of people with ED sourced from a general population sample of low- or middle-income countries. Thus, the present study aimed to assess whether there are differences in the composition of foods consumed during OBE in individuals with BESC sourced from the city of Rio de Janeiro, Brazil.

## 2. Materials and Methods

### 2.1. Participants and Procedures

The present study is part of the Binge Eating in Rio survey, an in-person, two-phase population-based household survey that assessed the prevalence of BED and its correlates among a representative sample of 2297 individuals from the population of the city of Rio de Janeiro. The study sample size was calculated in the planning phase according to the Brazilian Demographic Census [29] to assure confidence in the results (see Appolinario et al. [9] for further details on sampling procedures). Rio de Janeiro is the second largest city in Brazil, which is considered an upper-middle income country by the World Bank, as its Gross National Income (GNI) per capita in 2021 was between USD 4256 and USD 13,205 [30]. Eligible participants comprised adults aged from 18 to 60 years. Pregnant and lactating women were excluded. The data collection was performed in two stages between September 2019 and February 2020. In the first stage, all participants were interviewed face-to-face by trained lay interviewers. They also measured weight and height. In the second stage, participants who screened positive for BESC and a sub-sample of screen-negative cases were invited to answer a telephone interview for diagnosis confirmation [9].

### 2.2. Measures

#### 2.2.1. Diagnosis of BESC

In the first stage of the survey, the participants completed the Questionnaire on Eating and Weight Patterns (QEWP-5) for the screening of BESC (Table 1). QEWP-5 is a self-report instrument developed to screen BED and BN according to DSM-5 criteria [31]. The Brazilian version of the questionnaire was validated in a sample from the general population and showed satisfactory psychometric properties [32]. For the purpose of this study, RBE was identified when individuals reported OBE at least once a week in the last three months but did not fulfill the other criteria for BED (i.e., at least three of five binge eating-associated features and marked distress regarding the episode) [9].

In the second stage, a research assistant selected all screen-positive cases of BED and BN, and a subset of the screen-negative cases. They were interviewed by telephone by two Ph.D. students (Carlos Eduardo Ferreira de Moraes and Carla Mourilhe) experienced in ED assessment to confirm BED and BN diagnoses. The interviews were conducted according to the eating disorders section of Structured Clinical Interview for DSM-IV (SCID-P) [33], adapted to DSM-5. SCID-P is the gold standard method for the diagnosis of ED and was validated for administration by telephone [34]. All interviews were revised by a senior psychiatrist (JCA), experienced in the field of ED and blind to QEWP-5 answers. As detailed in Moraes et al. [32], at the end of the interviews, the interviewers and the psychiatrist followed the SCID-P questions discussing each symptom reported by the participants. If there was any disagreement regarding the diagnosis provided by the interviewers, this was discussed until consensus was reached.

#### 2.2.2. Food Consumption

Food consumption during a typical OBE in the previous three months was assessed by the trained lay interviewers in the first stage of the survey through the following question from QEWP-5: “*As best you can remember, please list everything you ate and drank during that episode. Please list the foods eaten and liquids consumed during the episode. Be specific—include brand names where possible and amounts or portion sizes as best you can estimate*”. Information about the amount of food consumed, cooking method (e.g., raw, cooked, or fried), and condiments or sugar/sweeteners added were also collected. This information was obtained through an instrument based on an offline software for 24 h food recall (ERICA-REC24h, available in www.erica.ufrj.br), validated for use in population-based epidemiological studies [35]. The interview technique used was the multiple-pass method, which consists of a five-step interview with the objective of reducing underreporting of food intake. This software used the list of foods from the 2008–2009 Brazilian Household Budget Survey [36], and it was adapted for the assessment of food consumption during the episodes. These strategies have been used to assess food consumption during binge eating episodes in ED research [37,38].

The food items reported were grouped into 13 categories according to their nutritional characteristics and their use in the Brazilian dietary pattern, as proposed by Pereira et al. [39]. Thus, the following groups were created: staple foods (rice and beans), fruits and vegetables, tubers, pasta and bread, fast foods (including snacks, pizza, and sandwiches), sweets and desserts (including chocolates), meats (red meat, pork, poultry, fish, and eggs), dairy products, alcoholic beverages, fruit juices, sugar-sweetened beverages, non-alcoholic beverages (including coffee and tea), mixed dishes (items that include ingredients from multiple categories, such as soups, pies, stir-fried dishes, and casseroles). In addition, for each BESC, the frequency (%) of consumption of each food group was estimated by the number of items reported in the food group divided by the total number of items reported by the participants with the respective diagnosis.

The Brazilian Table of Food Composition (TBCA) v.7.1 [40] was used to convert the reported food items into amounts of energy and macronutrients. TBCA describes nutritional data of Brazilian foods with the reliability ensured by the International Food Data System Network of the Food and Agriculture Organization of the United Nations (FAO) [41], which determines guidelines and criteria to be used in the generation, compilation, and use of food composition data.

#### 2.2.3. Sociodemographic and Metabolic Variables

Sociodemographic characteristics included age, sex, and ethnicity. They were obtained at the beginning of the household interview. Weight and height were measured at the participants’ house. Weight was assessed using a calibrated digital scale with a maximum capacity of 150 kg and a precision of 100 g (Plenna^®^, São Paulo, Brazil). Height was measured using a portable stadiometer with a maximum range of 200 cm and a precision of 0.1 cm (model 206; Seca^®^, Hamburg, Germany). Participants were weighed and measured while barefoot, wearing light clothing, with arms hanging alongside the body. BMI was calculated and classified into the following categories: underweight (<18.5 kg/m^2^), normal weight (18.5 kg/m^2^–24.9 kg/m^2^), overweight (25 kg/m^2^–29.9 kg/m^2^) and obesity (≥30 kg/m^2^) [42].

### 2.3. Data Analysis

Data are presented through weighted prevalence, means, standard error (SE), and 95% confidence intervals (95% CI). Information on the weighting approach is reported elsewhere [9]. First, analyses were stratified by sex and BMI, but there were no differences in the food consumption between men, women, and across BMI categories. Due to the complex survey design, the “PROC SURVEYFREQ” procedure from the Statistical Analysis System (SAS) was used to compare observed and expected weighted cell frequencies (prevalences) of demographics and metabolic characteristics. The differences were tested through the Wald chi-square test, as it considers the complex survey design. Between-group differences in the frequency (%) of intake of food groups were assessed through the Wald chi-square test. Furthermore, differences in the energy intake and the proportion of calories from carbohydrates, proteins, and lipids across ED status were assessed using the t-test. All statistical analyses were performed considering weights and the complex design of the survey through *Proc Survey* procedures in the SAS, release 9.5 (SAS, 2003). 

## 3. Results

### 3.1. Sample Characteristics

A total of 136 participants were diagnosed, 29 with BED, 17 with BN, and 90 with RBE. Participants with BED, BN and RBE had a mean age of 40.3 (SE = 3.3), 31.9 (SE = 3.7) and 34.7 (SE = 1.4) years, respectively. Regarding sex, all ED diagnosis were more prevalent in females (2.3% for BED, 1.3% for BN and 5.6% for RBE). Additional sociodemographic characteristics are reported in Table 2.

### 3.2. Food Preferences during OBE

Regarding individuals with BED, the most consumed food groups were staple foods (25%), chocolates/sweets/desserts (18.6%), meats (12.2%), fast foods (10.3%) and sugar-sweetened beverages (10.3%). In people with BN, chocolates/sweets/desserts (23.4%), sugar-sweetened beverages (18.7%), fast foods (15.6%), and meats (10.9%) were consumed more frequently. Participants with RBE also consumed more frequently chocolates/sweets/desserts (21.9%), staple foods (18.4%), and sugar-sweetened beverages (11.3%) during OBE. In the comparisons across diagnoses, staple foods were consumed more frequently by individuals with BED and RBE than those with BN (BED vs. BN: *p* = 0.01; RBE vs. BN: *p* = 0.04). In addition, the consumption of sugar-sweetened beverages was significantly more frequent in BN group than in BED group (*p* = 0.05) (Table 3).

### 3.3. Energy Intake and Macronutrient Composition of OBE

In the total sample, the mean energy intake during OBE was 1067 kcal. When splitting the results by ED status, individuals with BED consumed a mean of 1184 (95% CI 891; 1476) kcal. Participants with BN consumed an average of 1023 (95% CI 680; 1365) kcal. Finally, subjects with RBE consumed a mean of 994 (95% CI: 793; 1195) kcal. Regarding the macronutrient composition of the episodes, participants with BED consumed 59% (95% CI: 51; 67) of carbohydrates, 15% (95% CI: 12; 18) of proteins, and 28% (95% CI: 22; 35) of lipids. Subjects with BN consumed 56% (95% CI: 48; 64) of carbohydrates, 14% (95% CI: 7; 22) of proteins, and 32% (95% CI: 24; 39) of lipids. Individuals with RBE consumed 58% (95% CI: 55; 62) of carbohydrates, 13% (95% CI: 11; 15) of proteins, and 30% (95% CI: 27; 33) of lipids. The caloric intake and the macronutrient composition of the OBEs did not differ significantly across the ED diagnoses (Table 4).

## 4. Discussion

To our knowledge, the present study is the first to assess the composition of foods consumed during OBE in individuals with BESC from the general population of a metropolitan city in a middle-income country. Our results revealed that food consumption during OBE was different across the diagnoses. Individuals with BED and RBE consumed staple foods more frequently than those with BN during the episodes. In contrast, the consumption of sugar-sweetened beverages was more frequent by individuals with BN than by those with BED. Furthermore, we determined that the means of calory and macronutrient consumption during the episodes did not differ across groups. Overall, individuals with BESC consumed a mean of 1067 kcal, mostly from carbohydrates and lipids.

We determined that individuals with BED and RBE may have a different eating pattern from those with BN during OBE. While people with BED/RBE tend to more frequently consume dinner foods, such as rice, beans, and meats, participants with BN preferred foods with higher content of sugar and fat, such as chocolates, sweets, and sugar-sweetened beverages. However, as the body of literature in this regard has come to date from studies with clinical samples or laboratory test meals, the comparison with our results is limited. Overall, the findings suggest that individuals with BESC more frequently consume sweets, desserts, snacks, meats, breads, and cereals [23,24,25,26,27,43,44]. Specifically in Brazil, Alvarenga et al. determined women with BN frequently consumed sweets, desserts, crackers, and sugar-sweetened beverages during OBE [28]. Nevertheless, despite some similarities with our results, more studies are needed comparing the food selection across the diagnoses.

Our finding that there were no statistically significant differences in the energy intake during OBE across BESC was consistent with the results of our recent systematic review with a meta-analysis of studies with BED and BN subjects [20]. However, in the present study, the amount of calories consumed was lower than what has been reported in clinical settings (around 1800 kcal) [20]. OBE are characterized by the uncontrolled eating of excessively large amounts of food [2]. Nevertheless, the DSM-5 does not establish a minimum threshold of calories for such episodes. Overall, a cut-off of 1000 kcal has been used in the ED field as it seems to provide a good specificity in identifying OBE [45,46,47,48]. In this regard, previous research attempted to define an empirical threshold for a “large amount of food” and determined that these values may differ across food types and sexes [48,49,50]. For example, Forney et al. revealed that the upper limits for a “normal consumption” of foods ranged from 413 to 1074 kcal for women and from 466 kcal to 1611 kcal for men [48]. Taken together, these findings highlight that the mean OBE size reported in the present study is in line with what has been considered “a large amount of food” in the ED literature.

It is noteworthy that people who engage in regular OBE but do not meet the criteria for BED showed a similar eating pattern to those with full-threshold ED diagnosis (mainly BED). RBE is increasing in prevalence in both high and middle-income countries [13,15,51]. In addition, it has been associated with psychological distress, clinical and psychiatric comorbidities, poorer health-related quality of life (HRQoL), and functional impairment [9,13,15,51]. For example, in the Brazilian general population, data from the same participants of this study revealed that people with RBE had significantly higher rates of depression, anxiety, attention deficit hyperactivity disorder (ADHD), spine problems, and chronic muscle pain [9]. In addition, these participants showed significant impairment on HRQoL [9]. Thus, individuals with RBE could benefit from the inclusion of this condition into the usual clinical assessment of ED.

In the present study, the prevalence of BESC was significantly greater in females regardless of the diagnosis. In addition, BN was more prevalent in Black participants. Conversely, there were no differences in terms of age. These findings are consistent with those obtained by Udo et al., who discovered a greater prevalence of BED and BN in women from a large national sample in the United States [52]. Similarly, Solmi et al. reported that RBE was more common among females in Brazil [18]. Contrary to our findings, the authors reported a greater prevalence of RBE across younger and Black individuals [18]. Regarding BED and BN demographic correlates, although the lack of population-based studies assessing the prevalence of such conditions in Brazil limits the comparability of our results, previous research revealed that Black individuals were more likely to use laxatives [53]. Thus, future studies should assess the differences across BESC according to demographic characteristics.

Some potential explanations to our findings include: (1) Data from the Brazilian National Dietary Survey revealed that rice (84%) and beans (72.8%) are the most consumed foods in the country [54]. As OBEs in BED usually start as regular meals [28], we could expect that Brazilian staple foods would be frequently consumed during the episodes either. (2) Chocolates, sweets, and sugar-sweetened beverages are considered “forbidden foods” and likely to trigger OBEs due to their high energy density [55]. Thus, people with BN reduce the consumption of such foods during non-binge meals to limit the daily caloric intake and avoid weight gain [26,27]. Conversely, the consumption of chocolates, sweets, and snacks is greater during binge meals as the restrictive and purging type compensatory behaviors typical in BN patients may reduce avoidance of these foods [26,56,57,58]. In addition, liquids such as soda are commonly ingested during binge eating episodes to facilitate vomiting and reduce hunger [21,28]. (3) Food consumption is influenced by several cultural and socioeconomic characteristics, such as eating behavior, age group, sex, educational level, and income [59,60,61,62,63]. In this regard, data on dietary patterns of people with BESC originate mostly from high-income countries (e.g., USA) [20], which differ from Brazil in terms of traditional and contemporary eating practices [63]. For instance, in a comparison of the National Dietary Surveys from Brazil and USA, Bezerra et al. [64] reported that Americans consumed most food categories more frequently, with exception of meat, rice dishes, beans and legumes, spreads, and coffee and tea. (4) Data on food consumption during binge eating episodes originate mostly from laboratory-based studies which may not reflect subjects’ typical food consumption. In such contexts, people are provided with a single-food item (e.g., cookies) or a multiple-food item buffet. As these foods have a greater energy density, this could lead to a greater caloric intake than in studies with self-reported food recall [21].

Our results should be interpreted considering some limitations. First, the study sample is composed of individuals from the general population of the city of Rio de Janeiro, limiting the generalization of the results to urban settings. Second, participants could have underreported their food consumption due to embarrassment regarding the amount of food eaten during binge eating episodes [21]. However, the food recall is the most feasible self-report method to assess dietary intake [65]. Furthermore, it has been frequently used to assess food consumption in the ED literature [37,38]. Third, the great variability in energy intake, foods, and nutrients due to the use of only one food recall associated with the small number of cases may have underpowered the comparisons of food intake. The present study, however, has the following strengths: (1) the use of trained lay interviewers in the data collection; (2) the two-stage design to assess ED diagnosis, including the screening through the QEWP-5 and the use of SCID-P administered by clinicians specialized in ED assessment and treatment under the revision of a senior expert in this field.

Our findings have some clinical and public health relevance and may help healthcare professionals to treat BESC. For example, patients could benefit from nutritional counseling to increase the variety and the quality of foods consumed, providing a healthy and regular eating pattern that may help to reduce/eliminate binge eating episodes [66]. Further, although clinicians must be aware of the role of highly palatable and energy-dense foods, such as chocolates, sweets, and sugar-sweetened beverages as triggers for binge eating episodes, they should also encourage patients to consume such foods moderately, as a part of a balanced diet, reducing the eating restriction which may lead to overeating. In addition, people with BESC may benefit from a person-centered multidisciplinary care, involving psychiatrists, psychologists, and dietitians [66]. Finally, middle-income countries (e.g., Brazil) should develop public health strategies focused on nutritional education and healthy lifestyle, which could help decrease the clinical and social impairment of these conditions in this setting [67,68].

This study attempted to fulfill a gap in the literature regarding the dietary pattern of OBE in individuals with BESC in the general population. However, many aspects of binge eating episodes remain unclear. Thus, future research is needed to investigate (1) the food consumption and nutritional characteristics of subjective binge eating episodes—SBEs (when there is the feeling of loss of control over eating associated with the consumption of small or moderate amounts of food) [69]; (2) the nutritional composition of OBEs/SBEs in broader populations, different ethnicities, and in representative samples from other low- and middle-income countries; (3) the food consumption of individuals with other common ED (e.g., purging disorder); and (4) as detailed by Forbush et al. [21], further clarification of the validity of OBE size criteria, the duration and the frequency of the episodes are required to improve the accuracy of the assessment of BESC in both clinical and non-clinical contexts.

## 5. Conclusions

Eating patterns during OBE differed according to ED status in a metropolitan city in Brazil. Individuals with BED or RBE consumed dinner foods more frequently while people with BN preferred items with higher energy density and those easier to eat. Conversely, there were no differences in terms of caloric intake and macronutrient composition across the diagnoses.

Individuals with BESC from low- and middle-income countries may be at a high risk of clinical and functional impairment due to socioeconomic inequalities and the lack of access to specialized public health services. In this regard, providing multidisciplinary care delivered through multiple modalities (e.g., inpatient, outpatient, online interventions) and across different regions of Brazil would allow the earlier diagnosis and treatment of such conditions, reducing their economic and public health burden.

## Figures and Tables

**Table 1:** Diagnostic criteria for BED, BN, and RBE.

Criteria	BED	BN	RBE
OBE	Yes	Yes	Yes
Compensatory behaviors *	No	Yes	-
Frequency of OBE or compensatory behaviors	≥1 x/wk	≥1 x/wk	≥1 x/wk
≥3 of 5 binge eating associated features +	Yes	-	No
Marked distress regarding binge eating	Yes	-	No
Overvaluation of weight and shape	-	Yes	-

Note. OBE: Objective binge eating; BED: Binge Eating Disorder; BN: Bulimia Nervosa; RBE: Recurrent binge eating. * Self-induced vomit, excessive exercise, fasting, misuse of diuretics, laxatives, or other medications. + Eating faster than usual, eating until feeling disconformable full, eating large amounts of food when not feeling hungry, eating alone because feeling embarrassed by the amount of food eaten, feeling disgusted with yourself/depressed/very guilty. x/wk: times per week.

**Table 2:** Prevalence of binge eating spectrum conditions according to sociodemographic characteristics and weight status.

Variables	BED			BN			RBE		
	*n*	%	95% CI	*n*	%	95% CI	*n*	%	95% CI
Total	29	1.4	0.81–2.43	17	0.7	0.34–1.55	90	6.2	3.10–5.27
Sex									
Male	5	0.5 *	0.18–1.34	2	0.1 *	0.03– 0.62	19	2.4 *	1.40–3.99
Female	24	2.3	1.21–4.19	15	1.3	0.56–2.86	71	5.6	4.12–7.58
Race/skin color									
White	6	0.8	2.91–2.12	6	0.5	0.18–1.20	34	3.6	2.35–5.41
Black	7	2.4	0.91–6.31	6	1.8 *	0.56–5.90	18	4.2	2.15–7.90
Mixed ^a^	16	1.5	0.79–2.79	5	0.5	0.17–1.22	38	4.4	3.06–6.30
Age									
18 to 30 years	6	1.1	0.44–2.86	5	1.0	0.25–3.91	32	5.3	3.45–8.19
31 to 45 years	12	1.3	0.67–2.41	10	1.0	0.41–2.44	33	3.8	2.50–5.66
46 to 60 years	11	1.8	0.75–4.44	2	0.1	0.03–0.66	25	3.1	1.76–5.14
BMI ^b^									
Underweight	0	–		0	–	–	3	7.0	01.67–24.86
Normal Weight	1	8.1	1.08–41.64	2	7.7	1.60–30.06	16	18.2	10.56–29.51
Overweight	6	27.1	9.13–57.88	4	18.2	4.04–53.97	24	21.8	13.50–33.15
Obese	21	64.8 *	36.12–85.68	11	74.1 *	40.54–92.32	46	53.0 *	40.21–65.51

Note: * Statistically significant difference in the prevalence (*p* ≤ 0.05, Wald chi-square test for weighted frequencies); BED = Binge Eating Disorder (DSM-5); BN = Bulimia Nervosa (DSM-5), RBE = recurrent binge eating (≥1 binge eating episode/wk. in the last 3 mo.); ^a^ Mixed: Brown, Asian and Indigenous; ^b^ BMI = weight (Kg)/height (m^2^); results in bold are statistically significant at a *p* < 0.05.

**Table 3:** Frequency of food groups consumed during objective binge eating episodes according to eating disorder status.

Variable	Eating Disorder Status						*p*		
	BED (*n* = 29)		BN (*n* = 17)		RBE (*n* = 90)				
Total of food items consumed (*n*)	156		64		370				
Food groups	*n*	%	*n*	%	*n*	%	BED vs. BN	BED vs. RBE	BN vs. RBE
Staple foods (rice and beans)	39	25.0	5	7.8	68	18.4	0.01 *	0.51	0.04 *
Fruits and vegetables	7	4.5	2	3.1	21	5.7	0.23	0.85	0.73
Tubers	7	4.5	2	3.1	12	3.2	0.41	0.64	0.71
Pasta and breads	8	5.1	6	9.4	33	8.9	0.98	0.86	0.92
Fast foods (snacks, pizza, and sandwiches)	16	10.3	10	15.6	35	9.5	0.81	0.71	0.52
Chocolates, sweets and desserts	29	18.6	15	23.4	81	21.9	0.36	0.52	0.92
Meat, poultry, pork, fish and eggs	19	12.2	7	10.9	28	7.6	0.86	0.15	0.34
Dairy products	7	4.5	2	3.1	14	3.8	**	0.28	**
Soups and mixed dishes	1	0.6	1	1.6	2	0.5	**	**	**
Beverages	*n*	%	*n*	%	*n*	%	BED vs. BN	BED vs. RBE	BN vs. RBE
Fruit juices	2	1.3	1	1.6	8	2.2	**	**	**
Sugar-sweetened beverages	16	10.3	12	18.7	42	11.3	0.05 *	0.81	0.12
Coffee, tea, and other non-alcoholic beverages	2	1.3	0	0	11	2.3	**	**	**
Alcoholic beverages	0	0	0	0	5	1.3	**	**	**

Note. * Statistically significant difference at *p* ≤ 0.05 (Wald chi-square test); BED: Binge Eating Disorder; BN: Bulimia Nervosa; RBE: Recurrent binge eating; ** Differences were not tested due to the low frequencies in the groups.

**Table 4:** Energy intake and macronutrient consumption during objective binge eating episodes according to eating disorder status.

Variable	BED (*n* = 29)		BN (*n* = 17)		RBE (*n* = 90)		BED vs. BN		BED vs. RBE		BN vs. RBE	
	Mean (SE)	95% CI	Mean (SE)	95% CI	Mean (SE)	95% CI	*t* *	*p*	*t* *	*p*	*t* *	*p*
Energy intake (kcal)	1184 (145.5)	891–1476	1023 (170.5)	680–1365	994 (100.1)	793–1195	0.64	0.53	1.11	0.27	0.14	0.89
Carbohydrates (%)	59 (4.1)	51–67	56 (4.2)	48–64	58 (1.8)	55–62	0.49	0.63	0.15	0.88	−0.47	0.64
Proteins (%)	15 (1.3)	12–18	14 (3.7)	7–22	13 (1.0)	11–15	0.30	0.77	1.24	0.22	0.23	0.82
Lipids (%)	28 (3.3)	22–35	32 (3.7)	24–39	30 (1.5)	27–33	−0.54	0.59	−0.45	0.66	0.39	0.70

Note. * *t* test; BED: Binge Eating Disorder; BN: Bulimia Nervosa; RBE: Recurrent binge eating; SE: Standard error of mean; CI: Confidence interval.

## Data Availability

Data available upon reasonable request.
